# Ion Partition in Polyelectrolyte Gels and Nanogels

**DOI:** 10.3390/gels9110881

**Published:** 2023-11-07

**Authors:** Alexandros Chremos, Matan Mussel, Jack F. Douglas, Ferenc Horkay

**Affiliations:** 1 Section on Quantitative Imaging and Tissue Sciences, *Eunice Kennedy Shriver* National Institute of Child Health and Human Development, National Institutes of Health, Bethesda, MD 20892, USA; 2 Department of Physics, University of Haifa, Haifa 3103301, Israel; 3 Materials Science and Engineering Division, National Institute of Standards and Technology, Gaithersburg, MD 20899, USA

**Keywords:** gels, polyelectrolyte gels, ion partition

## Abstract

Polyelectrolyte gels provide a load-bearing structural framework for many macroscopic biological tissues, along with the organelles within the cells composing tissues and the extracellular matrices linking the cells at a larger length scale than the cells. In addition, they also provide a medium for the selective transportation and sequestration of ions and molecules necessary for life. Motivated by these diverse problems, we focus on modeling ion partitioning in polyelectrolyte gels immersed in a solution with a single type of ionic valence, i.e., monovalent or divalent salts. Specifically, we investigate the distribution of ions inside the gel structure and compare it with the bulk, i.e., away from the gel structure. In this first exploratory study, we neglect solvation effects in our gel by modeling the gels without an explicit solvent description, with the understanding that such an approach may be inadequate for describing ion partitioning in real polyelectrolyte gels. We see that this type of model is nonetheless a natural reference point for considering gels with solvation. Based on our idealized polymer network model without explicit solvent, we find that the ion partition coefficients scale with the salt concentration, and the ion partition coefficient for divalent ions is higher than for monovalent ions over a wide range of Bjerrum length ($l_{B}$) values. For gels having both monovalent and divalent salts, we find that divalent ions exhibit higher ion partition coefficients than monovalent salt for low divalent salt concentrations and low $l_{B}$. However, we also find evidence that the neglect of an explicit solvent, and thus solvation, provides an inadequate description when compared to experimental observations. Thus, in future work, we must consider both ion and polymer solvation to obtain a more realistic description of ion partitioning in polyelectrolyte gels.

## 1. Introduction

Ion partitioning is important in various separation membrane processes [1,2], chromatography [3,4], and ultrafiltration [5,6]. It also plays a key role in the formulation and controlled release of pharmaceuticals, food, and agricultural products [7,8,9,10]. In biology, it is a fundamental process utilized by living cells to control the electrochemical conditions within the intracellular space so that supramolecular edifices of charged macromolecules perform their biological function, such as cell–cell communication and signaling, osmotic stress response, muscle contraction, enzyme reactions, etc. [11,12,13,14]. Biological processes require an interplay between the dynamics of the enzyme reactions and the flux of mobile ions inside and outside the intracellular space, typically controlled by ion channels [12,15,16,17]. Moreover, ion specificity is important for protein solubility and water mobility change, as determined by the Hofmeister series [18,19,20,21,22]. While various model systems have been developed to mimic the response of living tissue to ions [23,24,25,26], a physically compelling model that allows for even a qualitative understanding of the physical principles governing these ion partitioning phenomena is needed. The present work is a preliminary study aimed at developing such a model.

In a crude approximation, a cell/intracellular space can be thought of as a polyelectrolyte gel, which consists of ionic-charged polymer chains bonded together to form a polymer network in an aqueous solution [27,28,29,30]. These gels exhibit many similar characteristics that cells exhibit in their response to changes in their environment, such as volumetric transition [31,32,33], ionic conductivity [34,35], and mechanical properties [36]. Discontinuous volume changes have received considerable attention because a small change in an intensive environmental variable, such as temperature or chemical potential, can trigger a large change in extensive properties, such as volume, and this promises a wide range of applications, such as drug delivery. It has been suggested that a biologically plausible mechanism for these gel transitions is a monovalent–divalent cation exchange [37,38,39]. However, important questions remain on the nature of the microscopic mechanisms by which discontinuous volume transitions take place. For example, how are the ions redistributed between the external bath and the gel phase before and after a volume transition?

The modeling of polyelectrolyte gels and, by extension, (poly)electrolytes in solution is a considerable challenge. Polyelectrolytes release their counterions into polar solvents, where they are dissolved [40,41,42]. This ionization process results in long-range repulsive Coulomb interactions between the polymer segments that cause the polymer to swell. The challenge lies in understanding how these interactions are influenced by counterions, which remain in the general proximity of the polyelectrolyte backbone [42,43]. This ionization process is complex, requiring an understanding of the competitive interactions between the various ionic species and the solvent. Indeed, in electrolyte solutions, where there is no polymer, the extent of ion-solvation plays a key role in both the trends in the solution viscosity of aqueous solutions and the origin of the Hofmeister series based on observations by Collins [44,45] and theoretical arguments by Salis and Ninham [46]. However, conventional modeling is often based on the ‘primitive’ model [47,48,49] and its various extensions of polyelectrolyte solutions [50,51,52], in which the explicit solvent degrees of freedom, and thus solvation effects, are completely neglected to simplify analytical and computational modeling. From a simulation perspective, the appeal of this type of modeling is that the solvent, the majority of the whole material, is treated *implicitly*. This idealized model of ionic and charged polymer solutions leads to significantly speeding up simulations of these complex solutions by greatly reducing the number of molecules that must be included in the simulations. From the standpoint of theory, this assumption leads to a drastic reduction in the analytic complexity that allows explicit analytic computations based on well-accepted mean-field models. However, these models of (poly)electrolyte solutions do not adequately address the solvation of the charged species, and several critiques exist in the literature [53,54,55,56,57]. Despite the reported shortcomings of this type of model, these models remain popular in the scientific community [58,59,60,61,62,63,64,65] and the performance associated with them in comparison with real polyelectrolyte gel materials remains uncertain.

In our previous studies, we have utilized an explicit description of the solvent, providing a direct way to modulate the competitive interactions between the oppositely charged species through solvation [66]. These studies indicated that the solvation of the charged species influences the spatial distribution of the counterions associated with the polyelectrolyte chains, leading to the ionization and localization of the counterions between the polyelectolytes. The nature of this localization can result in different kinds of ion and chain clustering through different mechanisms, such as chain depletion [67] or charge density waves [68]. Strong solvation of both polyelectrolyte chains and counterions can result in fractal-like clusters [69], which is uncharacteristic of the expected behavior of polyelectrolyte solutions based on primitive-type models. The solvation of ions in electrolyte solutions is also a key factor in reproducing the Hofmeister series [70]. Nevertheless, for the purposes of our study, we utilize an implicit solvent model as a zero-order approximation and a useful point of reference for future work.

In the present paper, we focus on understanding and quantifying the ion partitioning of monovalent and divalent ions induced by the presence of a polyelectrolyte gel structure. In particular, we study the monovalent and divalent ion partition of a hydro-gel structure immersed in single and mixed salt solutions at different ionic strengths and salt concentrations up to physiological conditions. We also consider sodium polyacrylate gels and compare them with model simulations based on molecular dynamics simulations of a coarse-grained bead-spring model. We directly calculate the ion partition coefficients ($Q_{i}$) by measuring the ion concentration inside and outside the gel structure. We also quantify the degree of swelling as the electrostatic environment changes. In gels immersed in a single ion valence salt, we find that divalent ions have higher ion partitioning than monovalent ions, meaning that the gel structure exhibits an affinity for the higher ion valence ions. Divalent ions decrease the gel’s swelling, resulting in more compact gels. The ion partition for both monovalent and divalent ions exhibits values of $Q_{i}$ comparable to experiments; however, the trends with the addition of divalent ions were inconsistent with experimental observations. We believe this inconsistency is due to an implicit description of the solvent without considering the effects of solvation.

## 2. Results and Discussion

We initiate our discussion by investigating gel structures immersed in a solution with a single salt type, i.e., either monovalent or divalent ions; the coions are monovalent of a negative charge in all cases. Subsequently, we will discuss gels in mixed salts with monovalent and divalent ions.

### 2.1. Ion Partition with One Ion Type

**Figure 1:**
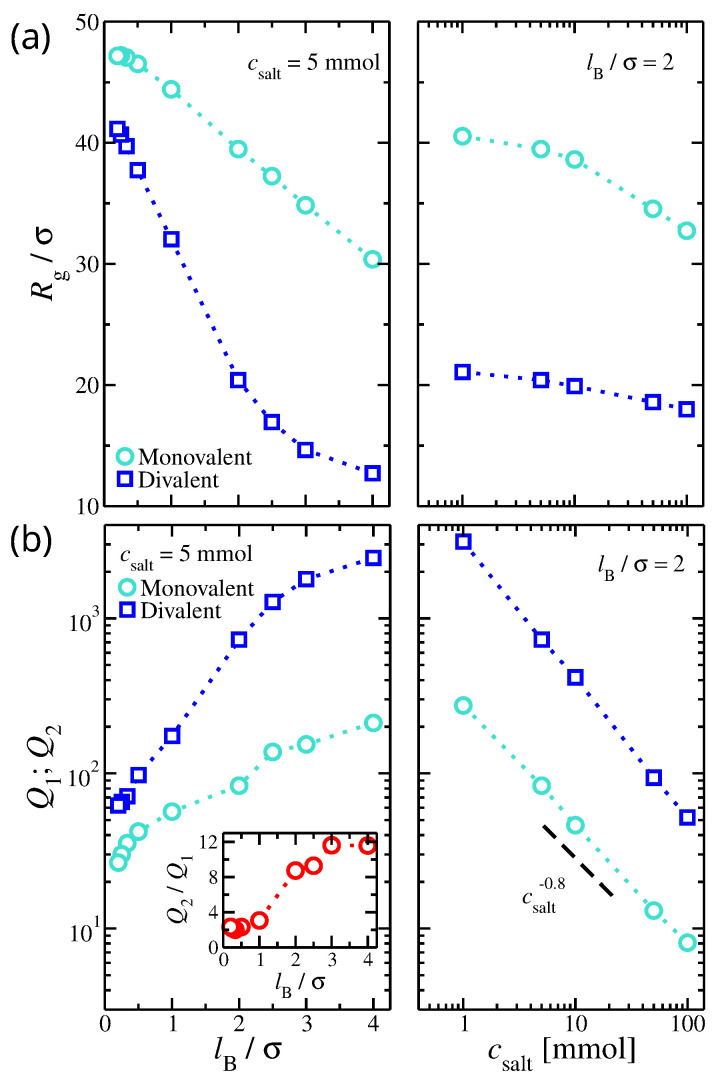
(**a**) Radius of gyration of the gel structure as a function of (**Left**) Bjerrum length, $l_{B}$, and (**Right**) the salt concentration, $c_{\mathrm{salt}}$. (**b**) The ion partition for monovalent, $Q_{1}$, and divalent, $Q_{2}$, ions as a function of (**Left**) $l_{B}$ and (**Right**) $c_{\mathrm{salt}}$. Inset: the ratio of $Q_{2}$ over $Q_{1}$ as a function of $l_{B}$. The results presented here are for gels immersed in a solution having a single type of ion valence, i.e., no mixed salt.

The size of the gel structure is influenced by the variation in the ionic concentration, ion valence, and strength of the ionic interactions. For weak Coulombic interactions (low Bjerrum length) and low salt concentrations, the gel structure is swollen due to the repulsive Coulombic interactions between the gel’s segments. Adding salt or decreasing the strength of electrostatic interactions (lower values of $l_{B}$) weakens these repulsion forces, resulting in a decrease in the gel’s radius of gyration, $R_{g}$ (see Figure 1a). The observed changes in gel size are more pronounced for solutions with divalent than monovalent ions since the adsorbed divalent ions force the chains composing the gel to “wrap” around them, leading to a further decrease in the persistence length [43] and a significant deswelling of the polyelectrolyte gels. The gel $R_{g}$ scales linearly with $l_{B}$ until the gel collapses into a nearly compact state, as was found for the case of divalent ions for $l_{B}/\sigma >3$. In aqueous conditions, divalent ions significantly reduce the size of the gel structure by a factor of two over a wide range of salt concentrations.

**Figure 2:**
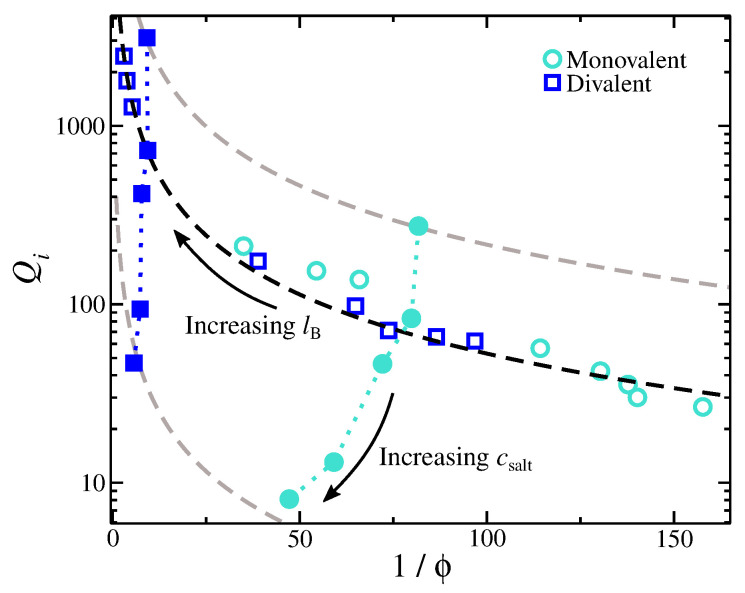
Ion partition coefficient, $Q_{i}$, for monovalent and divalent cases as a function of the degree of swelling, 1/ϕ, where ϕ is the volume fraction at the internal part of the gel. Open symbols correspond to gels immersed in a solution having 5 mmol of a single valence salt, and the variation in ϕ is due to variation in $l_{B}$. Filled symbols correspond to gels immersed in a solution having $l_{B}/\sigma =2$. The dashed lines are based on Equation (1) with κ≈1.1.

The ionic partitions, $Q_{1}$ and $Q_{2}$, calculated from independent simulations at the same salt concentrations, increase with $l_{B}$, and over a wide range exhibit a linear dependence (see Figure 1b). A possible interpretation is that the size of the gel structure is correlated with the ion partition coefficients since a dense gel structure has a higher negative charge density that attracts ions from outside the solution and is adsorbed inside the gel structure. Thus, any effect that may reduce the size of the gel structure also increases the ion partition coefficients. We use the gel’s volume fraction ϕ in the internal part of the gel as an alternative to $R_{g}$. As a zero-order approximation, we assume the following functional form,

(1) $$Q_{i}∼\phi^{\kappa }c_{\mathrm{salt}}^{-\mu },$$

where κ is a fitting parameter associated with the topology of the gel structure and the ionic environment, and μ is an empirical power-law exponent that characterizes the ion partition dependence on salt concentration. We find that the functional form of Equation (1) describes the scaling of $Q_{i}$ of gels having divalent ions rather well (see Figure 2). Monovalent ions also exhibit a relatively good agreement, though there are small deviations that require future investigation. Overall, the functional form of Equation (1) suggests a way to increase $Q_{i}$ by having a more compact gel. A more compact polyelectrolyte gel means a higher gel charge density, which attracts the mobile ions from the bulk into the gel structure.

On the other hand, $Q_{1}$ and $Q_{2}$ decrease with $c_{\mathrm{salt}}$. While an increase in salt concentration reduces the size of the gel structure, the effect is not as pronounced, suggesting that most added salt remains in bulk. Thus, the ion concentration in bulk increases faster than the ability of the gel structure to adsorb excess ions, leading to a sharp decrease in $Q_{1}$ and $Q_{2}$. Specifically, the fitted scaling exponent μ was μ≈0.76 and μ≈0.89 for monovalent and divalent ions, respectively (see Figure 1b). We find that the values of ion partition coefficients can vary by several orders of magnitude.

The ratio of the ion partitions $Q_{2}/Q_{1}$ for gels having a single ion valence in their solutions is approximately $Q_{2}/Q_{1}\approx 2$ for small values of $l_{B}/\sigma <1$. However, it increases rapidly for $l_{B}/\sigma >1$, reaching $Q_{2}/Q_{1}\approx 12$ for $l_{B}/\sigma =4$. For aqueous solutions ($l_{B}/\sigma \approx 2.1$), $Q_{2}/Q_{1}\approx 8$. An increase in the asymmetry between the ion partition coefficients is attributed to the strength of electrostatics having a more pronounced effect on divalent ions than on monovalent ions.

### 2.2. Mixed Ion Valence Salt

**Figure 3:**
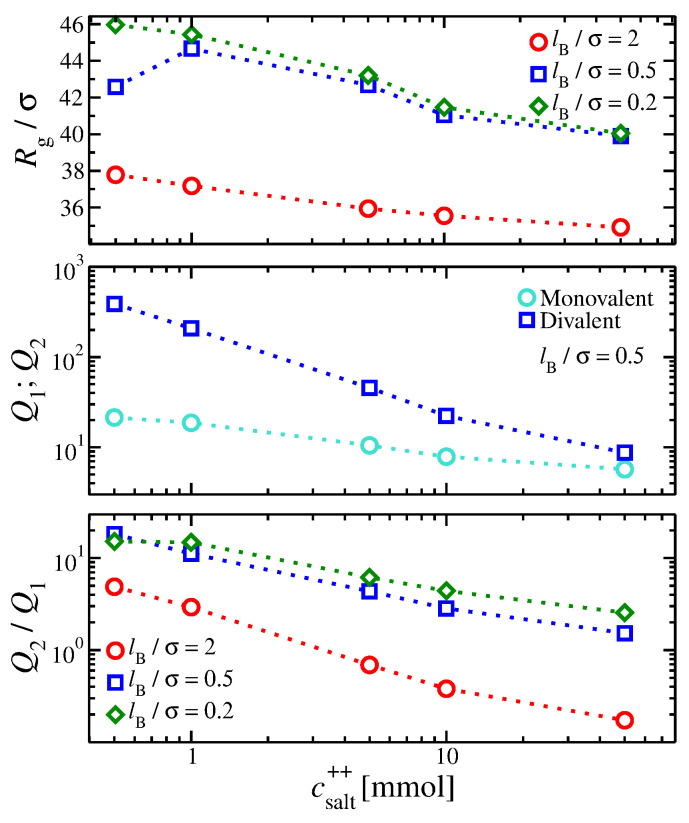
(**Top**) Radius of gyration of the gel structure, $R_{g}$, and (**Middle**) ion partition coefficients for gels containing both monovalent and divalent ions, $Q_{1}$ and $Q_{2}$, respectively, as a function of the divalent salt concentration. (**Bottom**) The ratio $Q_{2}/Q_{1}$ as a function of the divalent concentration is also presented.

Now, we examine the influence of having mixed salt solutions. We focus on having a gel structure in a solution having $c_{\mathrm{salt}}^{+}$ = 10 mmol excess monovalent salt and progressively increasing the concentration of divalent ions, $c_{\mathrm{salt}}^{++}$. In the mixed salt gels having $c_{\mathrm{salt}}^{++}\to 0$, we find that $R_{g}$ is approximately the same with gels having single monovalent ions (see Figure 3), suggesting that for low values of $c_{\mathrm{salt}}^{++}$, the gels behave as in a monovalent-type salt solution.

As $c_{\mathrm{salt}}^{++}$ increases, the gel size $R_{g}$ progressively decreases at a larger rate than if monovalent salts are added. The divalent ions inside the gel structure exert electrostatic forces that counter the swelling pressure originating from repulsive forces between the gel’s segments. However, the overall effect is small compared to having only divalent ions in the solution. Similar trends observed in gels having a single ion valence are found with the $l_{B}$ variation; compare Figure 1a and Figure 3.

Despite the relatively small changes in the size of the gel, we find significant changes in the behavior of the ion partition coefficients. Divalent ions prefer to reside within the gel structure since $Q_{2}>Q_{1}$. However, this difference decreases as $c_{\mathrm{salt}}^{++}$ increases. This trend deviates from gels having a single ion valence, where $Q_{2}$ was always higher than $Q_{1}$ for all the electrostatic conditions explored here. Evidently, divalent ions do not easily displace the monovalent ions from the gels in our current model of polyelectrolyte gels. While the pairwise divalent ion–gel segment interactions are enthalpically favored over the monovalent ion–gel segment interaction, the presence of monovalent ions, which repel divalent ions, inside the gel structure reduces the divalent ion favorability/selectivity. Higher values of $l_{B}$ reduce the asymmetry in the ion partition coefficients, suggesting that a stronger electrostatic interaction strength should reduce the preference for the divalent ions to localize within the gel structure (see Figure 3). Other factors beyond the ion valence are needed to enhance the divalent ion selectivity of the gel structure, such as the solvent quality and addressing the non-homogeneous spatial distribution of the dielectric.

**Figure 4:**
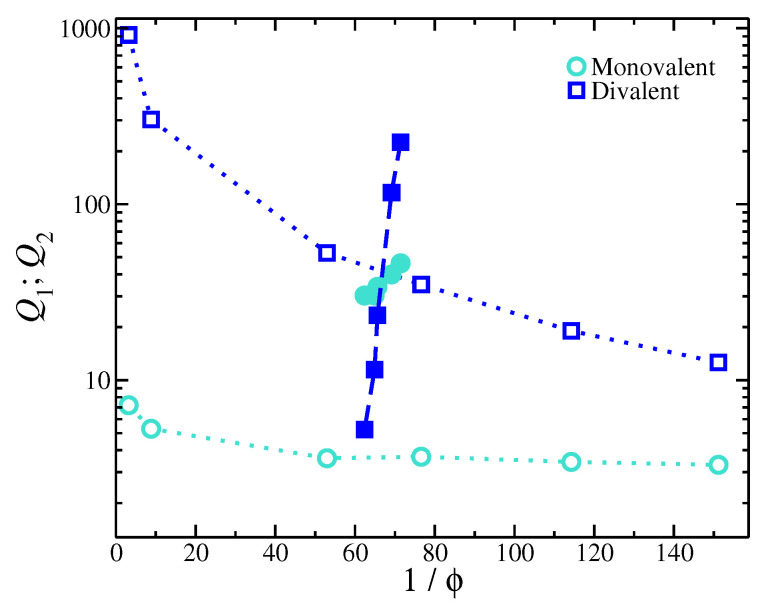
Ion partition coefficients for monovalent ($Q_{1}$) and for divalent ($Q_{2}$) ions for gels having mixed salt as a function of the degree of swelling (inverse of the volume fraction of the inner parts of the gel structure). Open and filled symbols correspond to experimental and simulation results, respectively.

In Figure 4, we present the ion partitions in sodium polyacrylate gels immersed in a mixed monovalent and divalent salt solution as a function of the degree of swelling. The addition of divalent salt significantly decreases the gel’s swelling. At the same time, $Q_{2}$ significantly increases, meaning that the interior of the gel becomes more favorable to divalent ions as the gel contracts. On the other hand, $Q_{1}$ is not influenced significantly by the gel’s volumetric changes. The measured ion partitions differ from the results obtained from the simulation model (see Figure 4). There are several possible reasons for this disagreement. We emphasize that, in the model, the ion and polymer solvation is neglected, and this might significantly influence this partitioning as the ion solvation can be expected to greatly influence the partitioning of ions into the charged polymer’s hydration layer. This physics is neglected in the present model calculations.

First, we have to consider the gel’s size and topology. We have performed simulations with gels with a molecular mass approximately one order of magnitude larger than the currently used ones. We did not find significant deviations in the values of $Q_{i}$ (not shown here), meaning that the gels used in the current study are large enough to capture the features of macrogels. A few defects also did not influence the values of $Q_{i}$ (not shown here), but the importance of the gel’s topology is out of the scope of the current study and warrants further investigation.

Second, a key assumption in the model is a uniform dielectric, meaning that the strength of electrostatic interactions is the same both inside and outside the gel. The experimental trends in Figure 4 appear to be similar to the variation in $l_{B}$ in terms of the $Q_{i}$ dependence on 1/ϕ, suggesting that the addition of divalent salt has a significant influence on the gel structure by altering the electrostatic conditions inside the gel structure. The observed trends of the ionic partition coefficients are similar to the trends for the gels with a single type salt with the $l_{B}$ variation, i.e., $Q_{i}∼\phi^{\kappa }$; also, compare Figure 2 and Figure 4. This implies that, as the gel contracts, there is a considerable change in the strength of the electrostatic interactions inside the gel (changes in $l_{B}$), which is, in turn, responsible for the trends in $Q_{i}$ observed experimentally. Thus, the internal part of the gel and the bulk solution must be modeled separately. This is another consequence of neglecting the solvent, which may also contribute to the failure of the present implicit solvent simulations.

Third, the competitive interactions between monovalent and divalent ions with the gel segments are described as the sum of excluded volume and Coulombic interactions, as expected from the primitive model. Thus, divalent ions are expected to have more favorable interactions within the gel structure based solely on pairwise electrostatic interactions. However, our simulation results show that divalent ions are not always favored inside the gel structure. The reason is that divalent ions also interact with surrounding, higher-numbered monovalent ions. The repulsive divalent–monovalent ion interaction may overcome the favorability of divalent ions based on pairwise electrostatic interactions. As discussed in the introduction, this treatment is insufficient to capture these competitive interactions once the solvent interaction with the differently charged species is also considered. Solvation influences the distribution of diffuse and interfacial ions around the polyelectrolyte backbone [42,43,67,68,69]. In the case of two types of ions, we anticipate that the strength of solvation may be different, contributing to the asymmetry in $Q_{i}$ observed experimentally. Although the disagreement between the model used and the experiments is “disappointing” at one level, this discrepancy points to the importance of ion and polymer solvation in ion partitioning.

## 3. Conclusions

We investigated the ion partition of polyelectrolyte gels using a coarse-grained bead-spring model with explicit ions suspended in an implicit solvent. We investigated gel structures immersed in a solution with a single type of ionic valence, i.e., either a monovalent or a divalent salt, and gels in a mixed salt solution. For gels in single-valence salt solutions, we find that higher values of the Bjerrum length and higher salt concentrations significantly decrease the size of the gel structure. These effects are more pronounced for divalent ions because they reduce the persistence length of the charged polymer chains composing the polyelectrolyte gel. We calculated the ion partition coefficients for these gels, and we found a significantly higher degree of sorption for divalent over monovalent ions inside the gel structure for the whole range of Bjerrum lengths and salt concentrations explored here.

For gels with mixed salt, divalent ions continue to exhibit preferential sorption over monovalent ions, but only when the electrostatic interactions are relatively weak. This is because the divalent ions fail to displace the monovalent ion from within the gel structure for stronger electrostatic interactions. While the results of our simulations apparently disagree with experimental observations, we attribute this disagreement to the use of implicit solvent models of the solvent. Such primitive models of electrolyte and charged polymer solutions fail to capture the effects of solvation that are apparently crucial with regard to the physics of ion partitioning. While these models are computationally more efficient due to lower computational costs, they are clearly inadequate to describe certain aspects of polyelectrolyte gels.

## 4. Methods and Models

### 4.1. Simulation Model

We employ a bead-spring model suspended in an implicit solvent, which was developed previously for studying the swelling behavior of nanogel particles [71]. This polymer model is based on the Grest–Kremer bead-spring polymer model [72], where each segment represents approximately a Kuhn segment, capturing a great range of polymer chemistries [73]. All the beads, polymer matrix segments, and ions are assigned the same mass *m*, size σ, and strength of interaction ε; we assign ε and σ the units of energy and length, respectively.

**Figure 5:**
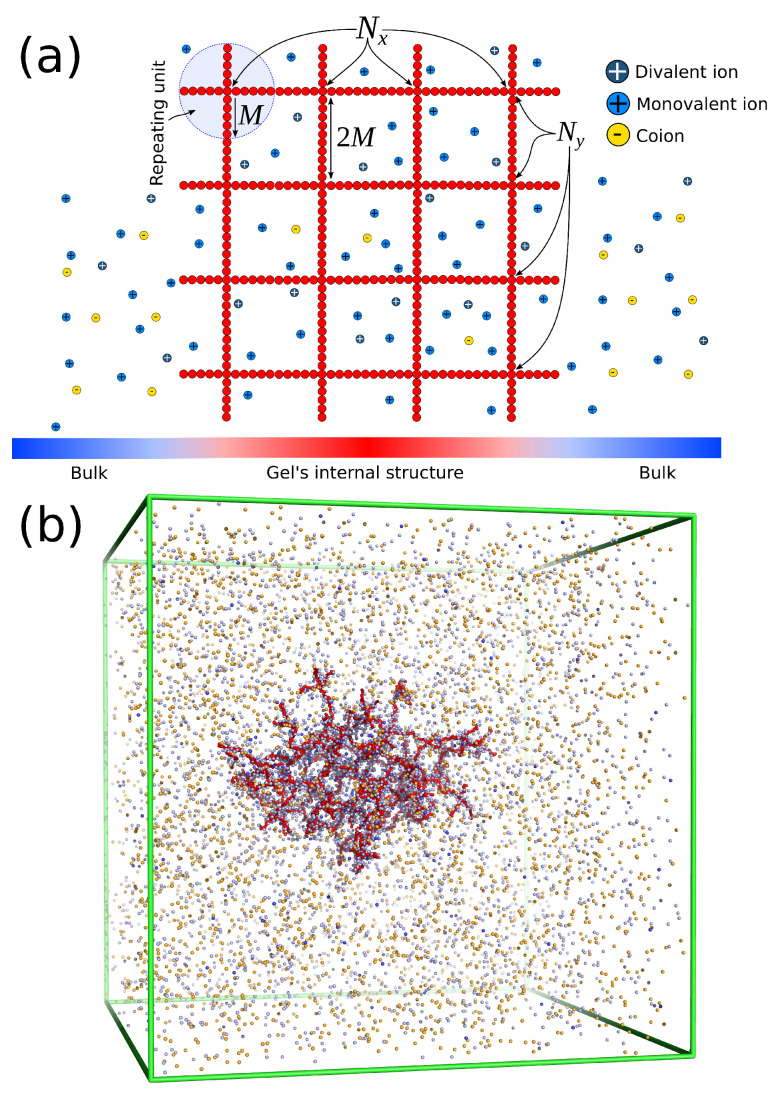
(**a**) Schematic of the molecular architecture of a finite size polyelectrolyte gel. The explicit description of monovalent, divalent, and coions inside the gel structure and in the bulk. (**b**) Typical screenshot of an equilibrated polyelectrolyte gel having f=4 and M=15 in mixed salt solution.

The polymer gel has the same construction as a “perfect compact gel” composed of star polymers placed in a square or cubic lattice with their free ends bonded with the free ends of the neighboring stars. The number of branched points (or star polymers) in each direction is labeled $N_{x}$, $N_{y}$, and $N_{z}$. The repeating branched structural unit of the polymer network studied here is identical to a regular star polymer. Other polymeric structures and/or other lattice morphologies could be utilized, but these are outside the scope of the current study. A regular star polymer has a core particle, which is connected with the free end of *f* chains (or arms) composed of *M* segments. Thus, the total number of interaction centers per star polymer is $M_{w}=fM+1$. The molecular mass of the polymer matrix is $M_{w,\mathrm{gel}}=\left(N_{x}N_{y}N_{z}\right)M_{w}$. We use the quantity $N_{b}$ to characterize the number of branched points in each direction. We focus on a polymer matrix having $N_{b}=N_{x}=N_{y}=N_{z}$ and f=4 arms. A schematic of the architecture of the polyelectrolyte gel and a typical molecular configuration is represented in Figure 5. We focus on gels having $N_{b}=4$, f=4, and M=15. The salt concentration is defined as $c_{\mathrm{salt}}=c_{0}\left(\rho_{+}+\rho_{-}\right)/2$, where $c_{0}$ is a conversion factor to real units, $\rho_{+}$ and $\rho_{-}$ are the number density of positive and negative charged ions, respectively.

The expression describing the interactions operating between all pairs of beads is the Weeks–Chandler–Andersen (WCA) potential [74], which is a Lennard–Jones potential cut and shifted at the position of the minimum, $r_{\min}=2^{1/6}\sigma$, to describe the purely repulsive interactions:

(2) $$V_{\mathrm{WCA}}\left(r\right)=\left\{\begin{array}{cc} 4\varepsilon \left[{\left(\frac{\sigma }{r}\right)}^{12}-{\left(\frac{\sigma }{r}\right)}^{6}\right]+\varepsilon \; & r\leq r_{\min}\\ 0\; & r>r_{\min} \end{array}\right.$$

This effective potential corresponds to athermal solvent conditions. Additionally, the effect of electrostatic interactions is described by a Coulomb potential [75],

(3) $$V_{\mathrm{Coul}}\left(r\right)/kT=l_{B}\frac{q_{i}q_{j}}{r_{ij}},$$

where $r_{ij}$ is the distance between beads with charge valences $q_{i}$ and $q_{j}$. The strength of the Coulomb potential relative to thermal energy ($k_{B}T$) is determined by the Bjerrum length $l_{B}=e^{2}/\left(\epsilon k_{B}T\right)$, where *e* is the elementary unit of charge and ϵ is the dielectric constant. For typical conditions in experimental aqueous solutions, $l_{B}^{\exp}\approx 0.71$ nm [76], and in reduced units, $l_{B}\approx 2.1\sigma$. The long range of the Coulomb potential is evaluated by the particle–particle–particle–mesh (PPPM) method [77,78] with an estimation accuracy of $10^{-4}$. All the polyelectrolyte gel’s segments carry a charge of single valence. Positively charged monovalent and divalent ions are also introduced into the system at specified concentrations. Monovalent co-ions are also introduced so that all the systems are electroneutral. The segments along a chain are connected with their neighbors via a stiff harmonic spring, $V_{H}\left(r\right)=k{\left(r-l_{0}\right)}^{2}$, where $l_{0}=0.99\sigma$ is the equilibrium length of the spring and $k=1000\varepsilon /\sigma^{2}$ is the spring constant.

Simulations were performed in a cubic box with length *L*; periodic boundary conditions were applied in all three directions. We utilized the large-scale atomic/molecular massively parallel simulator (LAMMPS) [79,80]. The systems were equilibrated at constant temperature, $k_{B}T/\varepsilon =1.0$, and constant pressure, maintained by a Nosé–Hoover thermostat [81,82,83,84]. Typical simulations equilibrate for 5000τ, and data are accumulated over a 150,000τ interval, where $\tau =\sigma {\left(m/\varepsilon \right)}^{1/2}$ is the MD time unit; the time step used was Δt/τ=0.005.

### 4.2. Experiments

Sodium polyacrylate gels were synthesized by free-radical copolymerization of sodium acrylate monomers in aqueous solutions containing 30% (*w*/*w*) monomers and 0.04% (*w*/*w*) N-N′-methylene-bisacrylamide crosslinker at 368 K. Ammonium persulfate (0.7 g/L) was used to initiate the polymerization reaction. After the reaction was completed, the gels were placed in deionized water to remove unreacted materials, such as sol fraction, and subsequently dried. A detailed description of the gel preparation process is given in Ref. [32].

Our gels were brought to equilibrium in an aqueous solution at room temperature, pH = 5.5, and containing salt of different concentrations. Afterwards, the gels were moved to new containers containing 3 mL nitric acid and 7 mL distilled water. The strong acid replaced the ions adsorbed onto the polymer chains, leading to gel deswelling as the ions were diffusing into the external solution. The ion content of nitric acid solution, now containing the ions from the gel, was measured with inductively coupled plasma optical emission spectrometry (ICP-OES). Control experiments were made to verify the accuracy of known samples and to correct for the cross-effects of having multiple ions and nitric acid in the solution. The ion concentration inside the gel was calculated by dividing the measured ionic content by the gel volume. Gel partitioning was calculated by dividing the ion concentration inside the gel by the ion concentration in the external bath solution.

### 4.3. Calculation of Ionic Partition Coefficients

**Figure 6:**
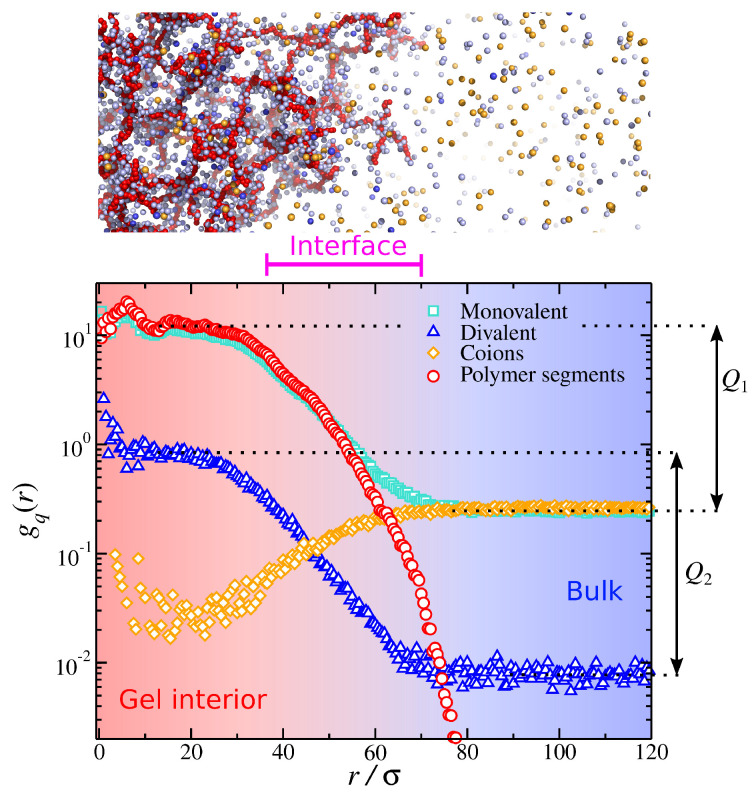
Charged particle pair correlation as a function of distance from the gel’s center of mass (r/σ=0) for a polyelectrolyte gel having a mixed monovalent and divalent salt. The partition coefficients $Q_{1}$ and $Q_{2}$ are also defined. A typical screenshot of gel is also presented, which approximately depicts the different ionic partitions identified in the figure.

We define the charged particle pair correlation $g_{q}(r)$ as the ratio of the local ionic particle concentration over the total (system-wide) ionic particle concentration as a function of distance from the gel’s center of mass. The quantity, $g_{q}(r)$, is akin to the pair correlation often used in the structure of soft condensed matter [85,86]. A representative example of polyelectrolyte gel in a mixed monovalent and divalent salt solution is presented in Figure 6. The presence of the polyelectrolyte gel influences the distribution of ions by creating different ionic concentrations inside ($c_{i,\mathrm{gel}}$) and outside ($c_{i,\mathrm{bulk}}$) the gel; the index *i* takes the values 1 and 2 corresponding to monovalent and divalent ions, respectively. To quantify this effect, we define the ion partition coefficient $Q_{i}$ as the ratio of the height of the plateaus of $g_{q}(r)$ inside and outside the gel structure (see Figure 6). This calculation is equivalent to the definition of the ratio of ion concentrations inside and outside the gel, i.e., $Q_{i}=c_{i,\mathrm{gel}}/c_{i,\mathrm{bulk}}$. From a typical simulation of a gel structure immersed in a mixed salt (see Figure 6), we identify three regimes based on the ion concentration. Away from the gel structure, we have the bulk, where the counterion concentrations exhibit a plateau. Near the center of the mass of the gel, we have the gel interior, which is characterized by a plateau in the counterion concentrations, and their values are higher than in the bulk. Finally, we find a crossover between the bulk and the gel’s interior at intermediate length scales, and this regime is associated with the gel’s interface.

## Data Availability

The data presented in this study are available from the corresponding author by reasonable request.
